# High Incidence of Tuberculosis, Low Sensitivity of Current Diagnostic Scheme and Prolonged Culture Positivity in Four Colombian Prisons. A Cohort Study

**DOI:** 10.1371/journal.pone.0080592

**Published:** 2013-11-21

**Authors:** Zulma Vanessa Rueda, Lucelly López, Lázaro A. Vélez, Diana Marín, Margarita Rosa Giraldo, Henry Pulido, Luis Carlos Orozco, Fernando Montes, María Patricia Arbeláez

**Affiliations:** 1 Grupo Investigador de Problemas en Enfermedades Infecciosas, Facultad de Medicina, Universidad de Antioquia, Medellín, Colombia; 2 Grupo de Epidemiología, Facultad Nacional de Salud Pública, Universidad de Antioquia, Medellín, Colombia; 3 Escuela de Microbiología, Universidad de Antioquia, Medellín, Colombia; 4 Sección de Enfermedades Infecciosas, Departamento de Medicina Interna, Facultad de Medicina, Universidad de Antioquia, Medellín, Colombia; 5 Facultad Nacional de Salud Pública, Universidad de Antioquia, Medellín, Colombia; 6 Secretaría Seccional de Salud y Protección Social de Antioquia, Gobernación de Antioquia, Medellín, Colombia; 7 Secretaría de Salud de Bello, Bello, Colombia; 8 Facultad de Enfermería, Universidad Industrial de Santander, Bucaramanga, Santander; 9 Secretaría de Salud de Medellín, Alcaldía de Medellín, Medellín, Colombia; Johns Hopkins Bloomberg School of Public Health, United States of America

## Abstract

**Objective:**

To determine the incidence of pulmonary tuberculosis (TB) in inmates, factors associated with TB, and the time to sputum smear and culture conversion during TB treatment.

**Methods:**

Prospective cohort study. All prisoners with respiratory symptoms (RS) of any duration were evaluated. After participants signed consent forms, we collected three spontaneous sputum samples on consecutive days. We performed auramine-rhodamine staining, culturing with the thin-layer agar method, Löwestein-Jensen medium and MGIT, susceptibility testing for first-line drugs; and HIV testing. TB cases were followed, and the times to smear and culture conversion to negative were evaluated.

**Results:**

Of 9,507 prisoners held in four prisons between April/30/2010 and April/30/2012, among them 4,463 were screened, 1,305 were evaluated for TB because of the lower RS of any duration, and 72 were diagnosed with TB. The annual incidence was 505 cases/100,000 prisoners. Among TB cases, the median age was 30 years, 25% had <15 days of cough, 12.5% had a history of prior TB, and 40.3% had prior contact with a TB case. TB-HIV coinfection was diagnosed in three cases. History of prior TB, contact with a TB case, and being underweight were risk factors associated with TB. Overweight was a protective factor. Almost a quarter of TB cases were detected only by culture; three cases were isoniazid resistant, and two resistant to streptomycin. The median times to culture conversion was 59 days, and smear conversion was 33.

**Conclusions:**

The TB incidence in prisons is 20 times higher than in the general Colombian population. TB should be considered in inmates with lower RS of any duration. Our data demonstrate that patients receiving adequate anti-TB treatment remain infectious for prolonged periods. These findings suggest that current recommendations regarding isolation of prisoners with TB should be reconsidered, and suggest the need for mycobacterial cultures during follow-up.

## Introduction

The incidence of tuberculosis (TB) in prisons is estimated to range from 25.3 to 6799 cases per 100000 prisoners per year, and the risk for TB is greater in prisons than in the general population [median estimated annual incidence rate ratio for TB: 23.0 (IQR: 11.7 – 36.1)][1]. In addition, the high incidence in prisons has a significant impact on the TB incidence in the community (median estimated fraction of TB in the general population attributable to the exposure in prisons for TB: 8.5% and 6.3% in high- and middle/low-income countries, respectively)[1].

TB is suspected when a patient presents with persistent productive cough for more than two weeks, which may be accompanied by other respiratory and/or constitutional symptoms[2]. Most clinicians and national and international guidelines use this criterion for TB screening, and only individuals meeting this criterion proceed with further work up for TB. A study conducted in Cambodia, Thailand, and Vietnam, on patients with HIV infection showed that the presence of a cough for two weeks or more had a sensitivity of 33% for TB. When those authors used the presence of cough of any duration in the preceding 4 weeks, the sensitivity increased to 71%[3]. Given the low sensitivity of the current recommendations, these results suggest that broadening the criteria for testing will result in an increased number of TB cases detected in high-risk prisoner populations.

Several risk factors reported in the literature (e.g., alcohol or drug users, or homelessness) may account for the high risk of TB in prisons, however, it is important to identify risk factors that can be used to stratify TB risk for screening purposes at the time of incarceration. 

In addition to identifying TB cases, in order to halt disease transmission, it is necessary to document when each TB case ceases to be infectious during treatment. This information can be used to guide health care providers in prisons on the optimal timing for discontinuation of respiratory isolation. The recommendations for discontinuation of isolation in prisons are as follow: 1) Treatment with a 4-drug regimen administered for at least 2 weeks by directly observed therapy (DOT); and 2) Clinical evidence of improvement in the condition of the inmate; and 3) Three consecutive negative sputum smears (obtained at least 8 hours apart, including one early morning specimen)[4]. Two factors must be taken into account before stopping isolation; the first is the time to sputum and culture conversion. One study reports that culture conversion from positive to negative in treated TB patients with fully susceptible disease is longer than was conventionally believed (median conversion time for culture: 38.5 days (95% CI, 33-43.5 days), 90^th^ percentile: 93 days)[5]. The second factor is whether the indication to end respiratory isolation should be based on sputum culture-negativity or sputum smear-negativity. A study from The Netherlands documented that 12.6% of the secondary TB cases were attributable to transmission from patients with smear-negative, culture-positive sputa, and similar rates of 17.3% to 22.2% were reported from Vancouver[6,7]. Therefore, it is important to clearly define when the patient is no longer infectious and is ready to leave respiratory isolation, while still avoiding the spread of TB. Clearly defined criteria are especially important in the context of inmates for the following reasons: most prisons are overcrowded, prisoners are in close contact with others for 14 hours or more per day inside the cells, living spaces within the cells are limited to less than 1 square meter per person, and the living spaces are poorly ventilated due to lack of windows and/or covering of windows by inmates [4].

The objectives of this study were to: a) determine the incidence of pulmonary tuberculosis in inmates in two male and two female prisons in two cities (Medellin and Bucaramanga) in Colombia; b) identify factors associated with active pulmonary tuberculosis, and assess the utility of epidemiological and clinical parameters in deciding who should be further investigated for TB; and c) determine the time to sputum smear and culture conversion after anti-TB treatment is initiated.

## Materials and Methods

### Study Design

Prospective cohort study. 

### Setting

Four prisons, in two cities, in Colombia (Medellín and Bucaramanga): Establecimiento Penitenciario de Mediana Seguridad Carcelaria de Medellín (prison capacity: 2,424), Complejo Carcelario y Penitenciario de Medellín Pedregal “COPED” Estructura I Medellín (prison capacity: 2,445), Establecimiento Penitenciario de Mediana Seguridad Carcelaria de Bucaramanga (prison capacity: 1,234), and Reclusión Mujeres Bucaramanga (prison capacity: 224). 

### Ethics Statement

The Ethics Committee of the School of Public Health (Facultad Nacional de Salud Pública) from the Universidad de Antioquia and the Instituto Nacional Penitenciario y Carcelario (INPEC) approved this research. The INPEC is responsible for the administration of the national prison system, and the security of prisons. Finally, the study was approved locally by the director of each prison where the study was conducted. All subjects included in the study signed two different written informed consents, one for TB and the other one for HIV diagnostic testing. All participants received pre- and post-test counseling. 

We gave the patients the written consent form and it was explained and signed in the presence of two witnesses. These witnesses were prisoners, and they had to sign the written consent form as well. 

For those who were not eligible for the study, we advised the health care service to start the clinical evaluation for other causes of respiratory symptoms, and they received the treatment according to the diagnosed disease. All people with lower respiratory symptoms that were invited to participate on the study agreed, and no one declined after signing the consent form. In fact, if an individual had recurrent symptoms and met the lower respiratory symptoms criteria, this person could re-enter the study. We had a separate written consent form to perform a HIV test. The form was also explained and signed in the presence of two witnesses. 

Information regarding participants was not shared with jail authorities, other prisoners, or family. We did not offer any payment for participating in the study.

The TB and HIV informed consent forms (Spanish originals) which were used in the study and approved by the Ethics Committee are attached.

As we studied active pulmonary TB, the people with extrapulmonary TB were diagnosed and treated by the health care service. All people, whether they entered the study or not, received the same access to health care services and treatment when needed.

All documents containing information that could identify a participant were password- protected. All data forms were coded and cleared of any personal information that could identify the inmate. We notified each participant about the TB and HIV test results. During the study period, we provided the only screening and laboratory testing for TB, individuals diagnosed with TB and/or HIV, were notified as was healthcare service of the prison in order to facilitate initiation of anti-TB treatment, and the antiretroviral treatment when it was indicated. The TB treatment administered was according to recommended regimen in Colombia (for susceptible cases it consisted of a combination of isoniazid, rifampicin, pirazinamide, and ethambutol for 8 weeks, and isoniazid, plus rifampicin for 4 months). If the *Mycobacterium tuberculosis* isolate was resistant to one or more drugs, infectious disease physicians from the Universidad de Antioquia evaluated the history of the patient, and made specific recommendations for treatment and follow-up. We conducted contact tracing for all inmates that had been in contact with a prisoner diagnosed with active TB. In addition, we asked for the names, phone numbers, and addresses of people that visited the prisoner, and sent the information to the State Health Authority to guide contact tracing investigation outside of the prison system. Finally, we followed all biosafety procedures according to international guidelines. The field team was evaluated at baseline, and every subsequent year with the tuberculin skin test (TST) to detect latent tuberculosis infection. No members of the study team had a TST conversion during the study.

### Eligibility Criteria

All prisoners older than 18 years of age, with respiratory symptoms (RS) of any duration. We studied for TB all people with lower respiratory symptoms that were identified during the study period.

### Selection of Participants and Procedures

The field team (three nurses, one physiotherapist and two physicians) visited the prisons from Monday to Friday (cells and corridors) between April 30, 2010 and April 30, 2012 in Medellin, and between April 30, 2010 to April 30, 2011 in Bucaramanga, and with the help of prisoners actively screened for prisoners with respiratory symptoms. After each participant signed the proper consent forms, one blood sample was taken for HIV testing, followed by western blotting if the screening test was positive. Once an HIV infection was confirmed, CD4 cell counts were obtained. Three sputum samples were taken on consecutive days in the presence of the field team. When the patient could not produce sputum, but he/she had weight loss or lymphadenopathy, sputum was induced. All three sputum samples were processed using the conventional sodium hydroxide-N-acetyl-L-cysteine method, with standard decontamination, and concentration methods. A smear was prepared for auramine-rhodamine staining to visualize acid-fast bacilli (AFB). The first sputum sample for each inmate was inoculated in Lowenstein-Jensen (LJ) medium, in a mycobacterial growth indicator tube (MGIT) incubated in MGIT 960 BACTEC instrument (BD Diagnostics, Sparks, MD, USA), and in thin-layer agar (TLA) for the detection of resistance to rifampicin and isoniazid as previously reported[8]. *Mycobacterium tuberculosis* was identified by standard biochemical tests. Susceptibility tests for first-line drugs were also performed on 7H11 Middlebrook agar using the proportion method with the following concentrations of antibiotics: 0.2 and 1.0 μg/ml isoniazid, 1.0 μg/ml rifampicin, 2 μg/ml streptomycin, and 7.5 μg/ml ethambutol. All antibiotics were purchased from Sigma-Aldrich, Co. (St. Louis, MO, USA). In the case of persistent symptoms after a negative TB evaluation, each patient consulted the health care service to discard other causes of cough.

### Follow-up

All prisoners with TB diagnosed by sputum smear or culture were followed for two years or until the end of the study from April 30, 2010 to December 23, 2012 in Medellin, and from April 30, 2010 to June 30, 2011 in Bucaramanga . Follow up visits were: monthly for six months after starting anti-TB treatment or for nine months in the case of HIV coinfection or monoresistance. Subsequently, follow up occurred on a bimonthly basis for the next six months and quarterly during the second year. During each follow-up, three sputum samples were taken and processed as previously outlined. The field team visited patients transferred to other prisons to complete the follow up. When patients were released, follow-up visits were set up at the Universidad de Antioquia or at their homes, according to each patient’s preference. A physician and a nurse evaluated the patient at diagnosis, and during all follow-up visits, wherein they completed a physical exam and recorded any adverse event related to the treatment.

### Variables

The following information was collected for all individuals with lower respiratory symptoms: age; history and time of prior incarceration; use of drugs (inhaled, injected, or smoked) or alcohol, along with the quantity and time of consumption; comorbidities (chronic obstructive pulmonary disease, diabetes, chronic kidney disease, HIV, and any other immunosuppressive disease); contact with a TB case (outside and/or inside the prison); history of prior TB, including date of last episode, treatment and outcome; risk factors for multidrug-resistant TB (MDR-TB); history of latent TB infection; prior administration of anti-TB drugs; cough; expectoration; hemoptysis; fever; and weight loss. To determine previous exposure to the BCG vaccine, the scar was explored in all included prisoners by a nurse on the field team. To determine overcrowding conditions, the width and length of the cells and corridors, and the number of prisoners in each cell were measured. During follow-up, patients were asked about respiratory symptoms as well as treatment adherence and any adverse event related to the treatment. 

### Definitions

Person with lower respiratory symptoms: A person who presents a cough of any duration and/or expectoration, and abnormal breath sounds on lung auscultation.

Case of active TB: A patient with *M. tuberculosis* complex identified from a sputum specimen by culture, or the presence of at least one acid fast bacillus (AFB+) in 100 immersion fields in at least one sputum sample plus lower respiratory symptoms and clinical evidence of improvement in the condition of the inmate after the initiation of anti-TB treatment, or both.

Smear-negative pulmonary TB case: Sputum that is smear-negative but culture-positive for *M. tuberculosis*.

TB contacts[9]: Persons who share air for prolonged periods of time with an active TB case. These include the following: 1) all prisoners who sleep in the same cell as a TB case; 2) prisoners who spend time in closed or poorly ventilated work areas (e.g., carpentry and handicraft shops, garment factories, bakeries) inside the prison; 3) prisoners who interact with a TB patient during recreational activities (e.g., playing cards or chess, watching TV); 4) prison staff who come in contact with a TB case; and 5) visitors.

Secondary TB case: TB contacts diagnosed with active TB.

Cure: A patient with active tuberculosis, who completed treatment, and who was smear- and culture-negative on two occasions, one of which was during the last month of treatment.

Default: A patient whose treatment was interrupted for 2 consecutive months or more.

Death: A patient who died for any reason during the course of treatment.

### Statistical Methods

An information systems expert and the statistician reviewed all data collection forms at baseline and after each follow-up to ensure data quality and completeness. If inconsistencies or missing data were identified, the field team was instructed to collect those data. Before the analysis, we randomly selected 131 participants from the entire pool, and reviewed the data collection form and the information entered into the database to evaluate any typing errors. Data was entered into a Microsoft Access® database, and statistical analysis was performed using SPSS version 20.0 (SPSS Inc., Chicago, IL, USA) and STATA 11.1 (StataCorp, College Station, TX, USA).

The TB incidence rates were estimated for all prisons and by city. The numerator was the number of positive cases by sputum smear and/or culture, and the denominator was the maximum number of people that were confined in the four prisons during the study period. Baseline characteristics are described as absolute and relative frequencies for qualitative variables. The age and time to sputum smear and culture conversion after anti-TB treatment initiation are reported as median with interquartile range (IQR). Living space, in square meters per person is reported as media ± standard deviation (SD) because this parameter had a normal distribution. The prevalence of HIV was estimated by the combination of positive cases enrolled in the study, and new cases diagnosed during the study.

To determine the factors associated with pulmonary TB, the cohort was divided into two groups: those with TB (cases) and those without TB (controls). The outcome was a positive result by sputum smear and/or culture. Initially, we performed a bivariate analysis to estimate the Odds Ratio (OR) and the 95% confidence interval (CI). Any of the following variables that had a p-value <0.25 were included in a binary logistic regression: age (≤24 years, >24 years), marijuana use (never, past, current), history of prior TB (no, yes), contact with a TB case (never, no household contact, outside the prison, inside the prison), and body mass index (underweight: <18 kg/mt^2^, normal: 18-25 kg/mt^2^, and overweight: >25 kg/mt^2^). We adjusted with generalized estimating equations (GEE) because reclusion within a cell block inside each prison is a shared condition that may influence the presence of TB. The model was estimated using the logit link function, binomial probability distribution and exchangeable correlation matrix. The OR and 95% CIs are reported and adjusted for age, use of smoked drugs, prior TB history, contact with a TB case and body mass index. We did not find significant differences between the results in the four prisons assessed, and therefore only reported global results.

To evaluate the time to sputum smear and culture conversion from positive to negative during anti-TB treatment, Kaplan-Meier curves were plotted. Time zero was defined as the start of anti-TB treatment, and the outcome was a negative result by sputum culture or smear. We censored patients lost to follow-up. The median time of culture conversion and the 95% CI were estimated for all patients with TB confirmed by culture at diagnosis, and were stratified by the result of sputum smear at baseline (negative, 1+, or 2+ or 3+). 

## Results

### Incidence and Baseline Characteristics

The maximum number of people held in four prisons during the study period was 9,507. Among them, 4,463 (46.9%) were screened, 2,103 (22.1%) were evaluated because they reported respiratory symptoms of any duration, and 798 (8.39%) were excluded because they did not have lower respiratory symptoms. The cumulative prevalence of inmates with lower respiratory symptoms was 13.7% (1,305/9,507). 

Of the 1,305 people who participated in the study, 54 were enrolled twice. Among them, five patients had persistent cough, one at the second month after the first entry to study, two at the third month, one at the fourth month and one at the fifth month. The other 49 patients did not have persistent cough and were evaluated for the presence of new symptoms. The difference between the first evaluation and the appearance of new symptoms for the second episode was: median: 183 days, IQR (89 - 321). 

**Figure 1:**
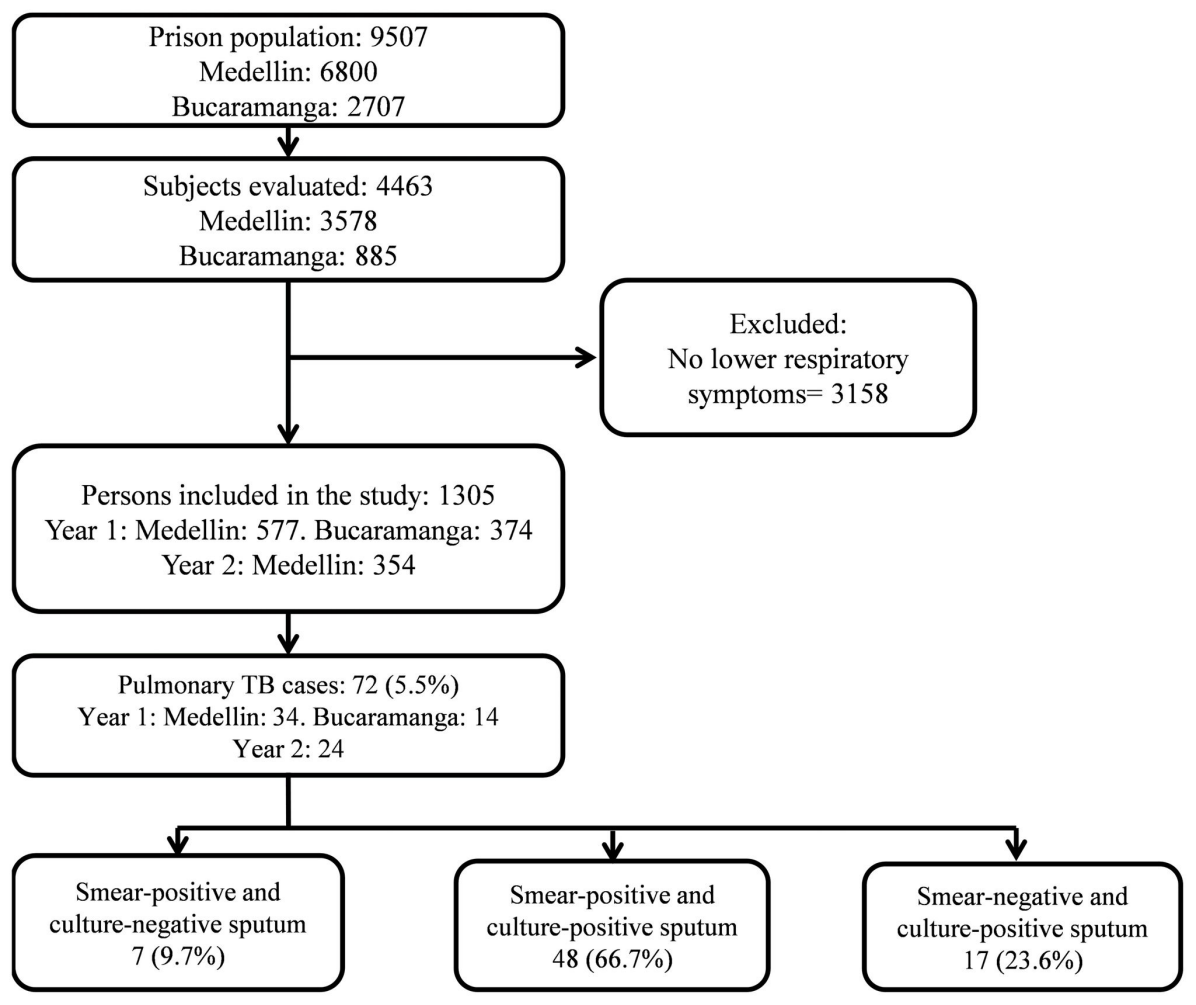
Flowchart of inmates included in the study.

Among the 1,305 included patients, tuberculosis was diagnosed in 72 cases (5.5%) (Figure 1), one TB case was diagnosed in a correctional officer. Two patients which had tested negative in the first entrance sample, were diagnosed with TB in the samples taken during the second enrollment.

The cumulative incidence rate during the first year was 505 cases per 100,000 inmates, and stratified by city, the cumulative incidence was 500/100,000 inmates in Medellin and 517/100,000 inmates in Bucaramanga. The cumulative incidence rate during the second year in Medellin was 353 cases per 100,000 inmates. 

The mean of living area per person was 1.28 ± 3.05 m^2^; no differences were found among prisons, or between the TB cases and symptomatic respiratory groups (table 1). The maximum prison populations imprisoned at the time of the study were as follow: prison 1: 6,000, prison 2: 800, prison 3: 2,409, prison 4: 298, and the percentages of overcrowding in terms of the difference of the facility people capacity (prisons capacity and current prison population) were: 147.5%, -68%, 95%, and 33%, respectively. 

**Table 1:** Baseline characteristics of prisoners with and without pulmonary tuberculosis at four prisons in Colombia, 2010-2012.

**Variables**	**Pulmonary TB cases (n=72)**		**Non-TB cases (n=1233)**	
**Age (years), median, IQR**	30	25 - 37	30	24 - 40
**Living space per person (m^2^**)**,** mean ± SD	1.46	2.59	1.28	3.08
**Time of incarceration (months), median, IQR**	12	5 - 27	8	3 - 16
**Time with cough (days), median, IQR**	15	14 - 90	15	14 - 40
	Frequency	%	Frequency	%
**Male sex**	63	87.5	1128	91.5
**Respiratory symptoms <15 days**	18	25.0	362	30.9
**History of** prior **incarceration**	23	32.4	449	36.5
**History of treatment for LTBI**	1	1.4	10	0.8
**Contact with a TB case**	29	40.3	334	27.1
**History of prior TB**	9	12.5	42	3.4
HIV positive status*	3	4.2	23	1.9
**Current drug use** ^†^	36	50	574	46.6
**Smoked (marijuana)**	36	50	574	46.6
**Inhaled**	14	19.4	206	16.7
**Injected**	0	0	1	0.1
**Comorbidities**	21	29.2	368	29.8
**Body Mass Index**				
**Normal**	54	75	890	72.2
**Underweight**	12	16.7	63	5.1
**Overweight**	5	6.9	236	19.1
**Cough**	72	100	1230	99.8
**Expectoration**	70	97.2	1224	99.3
**Fever**	47	65.3	587	47.6
**Weight loss**	41	56.9	483	39.2
**Hemoptysis**	18	25	148	12
**BCG scar**	60	83.3	1036	84.4

IQR: interquartile range. SD: standard deviation. LTBI: latent TB Infection. BCG: Bacillus Calmette-Guérin.

* 67 patients refused HIV testing. ^†^ Marijuana and cocaine.

The median duration of cough was 15 days (IQR 14 - 40), but 25% of 72 patients diagnosed with TB had less than 15 days of cough. Among TB cases, 40.3% (29/72) had a history of contact with a TB case, of which 48.3% (14/29) were inmates. Among the 12.5% of patients with a history of prior TB, the mean of the time of the last episode was 1.67±1.0 years. In this study, the prevalence of HIV was 2.1% (26/1238) overall and 4.2% among individuals with active TB; 67 persons declined HIV testing (table 1). 

### Factors Associated with TB

The multivariate analysis showed that the risk factors associated with active pulmonary TB were history of prior tuberculosis, contact with a TB case (either outside or inside the prison), and being underweight. Being overweight was a protective factor for active pulmonary TB (table 2). Living space per person as a measure of overcrowding was not included in the multivariate analysis because this variable was a constant for all prisons and patients. 

**Table 2:** Bivariate and multivariate analyses of factors associated with active pulmonary tuberculosis in prisoners in Colombia.

TB case	Crude Odds Ratio	95% CI	Adjusted Odds Ratio^*^	95% CI
**Age (≤24 years) (ref)**				
**Age (>24 years)**	1.31	0.75 - 2.29	1.43	0.80 - 2.56
**No marijuana user (ref)**				
**Past marijuana users**	2.29	1.16 - 4.51	1.70	0.83 - 3.42
**Current marijuana users**	1.73	0.93 - 3.23	1.47	0.75 - 2.83
**History of prior TB**	3.81	1.75 - 8.29	3.05	1.37 - 6.74
**No contact with a TB case (ref)**				
**No household contact**	1.04	0.50 - 2.17	0.95	0.45 - 1.99
**Contact with a TB case in the community (outside the prison**)	2.89	1.43 - 5.83	2.49	1.21 - 5.10
**Contact with an inmate with TB**	2.46	1.14 - 5.31	2.10	0.96 - 4.55
**Normal weight (ref)**				
**Underweight (BMI <18 kg/m^2^)**	3.07	1.60 - 5.88	2.92	1.50 - 5.68
**Overweight (BMI >25 kg/m^2^)**	0.35	0.14 - 0.86	0.39	0.15 - 0.98

* Adjusted for all other variables listed.

Ref: reference category; BMI: Body Mass Index

### Diagnosis and Time to Sputum Smear and Culture Conversion

**Figure 2:**
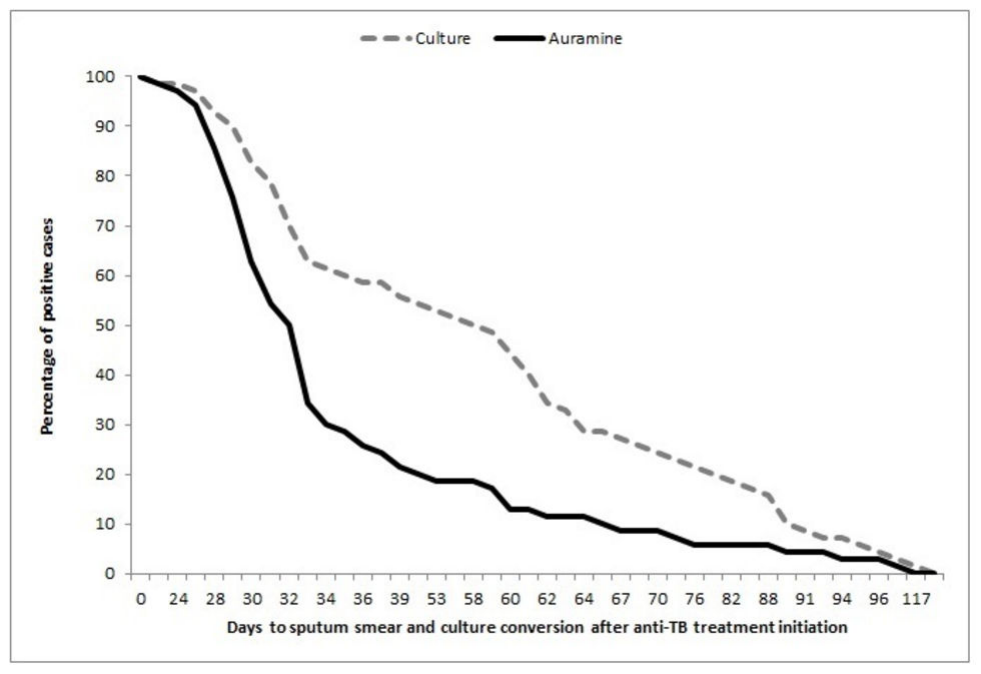
Time to sputum smear and culture conversion after anti-TB treatment initiation.

**Figure 3:**
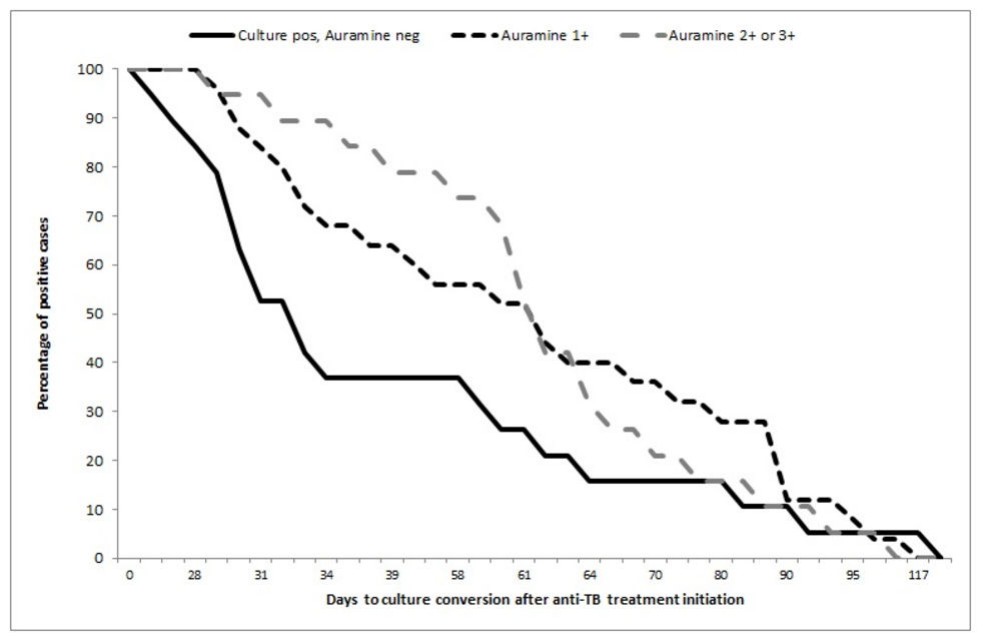
Time to culture conversion during TB treatment, stratified by sputum smear status at diagnosis.

A total of 23.6% (17/72) of pulmonary tuberculosis cases were diagnosed only by culture, and there were seven cases (9.7%) with smear-positive but culture-negative sputum. There were 18 cases of TB amongst 380 individuals with fewer than 15 days of symptoms (4.73%), and 54 cases amongst 925 people with 15 or more days of symptoms (5.83%), p-value: 0.43. Three cases (4.17%) were resistant to isoniazid, and two cases (2.78%) were resistance to streptomycin. There were no patients with MDR or XDR-TB. The median time to culture conversion during the anti-TB treatment was 59 days (IQR 32-70), and the median sputum smear conversion time was 33 days (IQR: 30-38) (Figure 2). The 90^th^ percentile value for culture conversion was 90 days, and 66 days for sputum smear conversion. Culture conversion was faster when the sputum smear at diagnosis was negative (median: 33 days) than when the sputum smear was rated 1+ or either 2+ or 3+ (median for both categories: 62 days) (table 3 and Figure 3).

**Table 3:** Time to sputum culture conversion during TB treatment, stratified by sputum smear at diagnosis.

Diagnosis of TB at baseline	Median (days)	95% confidence interval	
**Global**	60	56.7	63.3
**Culture positive with smear negative**	33	29.9	36.2
**Smear 1+**	62	47.4	76.6
**Smear 2+ or 3+**	62	60.3	63.7

### Follow-up

At the end of TB treatment, 88.9% (64/72) of patients were cured, 6.94% (5/72) had unknown treatment outcome because they were released and lost to follow-up, 2.78% (2/72) discontinued treatment (default), and 1.39% (1/72) died. None of the patients had an adverse event that necessitated discontinuation of anti-TB treatment. The patient who died had HIV and disseminated TB. During the two year follow-up, two patients (2.78%) relapsed, as determined by smear- and culture-positive sputum. Both patients had fully susceptible strains and were successfully treated by the end of their second course of anti-TB treatment.

When the State Health Authority completed contact tracing outside the prisons, three additional cases were identified among household members. Those cases were three family members that visited two prisoners diagnosed with TB on a weekly basis.

## Discussion

This study yielded the following main findings: 1) a high incidence of TB in prisons; 2) a low sensitivity of persistent cough, which underscores the importance of evaluating for TB in immunocompetent people with a cough of any duration and using culture methods for diagnosis; and 3) a long interval between treatment initiation and conversion to negative sputum by culture, which has implications for the duration of respiratory isolation in confined places such as prisons. 

In Colombia, one report of TB incidence from a prison in Pereira[10] documented a lower incidence than that found in this study (299 cases/100,000 prisoners in 2010 and 213 cases/100,000 in 2011). The difference may be attributable to real difference in incidence of TB and/or the methods used. Castañeda-Hernández, et al.[10] used Ziehl-Neelsen staining and one solid culture in Ogawa-Kudoh medium to diagnose TB, whereas we used auramine-rhodamine staining and three cultures methods, including a liquid medium. Thus, the higher incidence in the present study may be due to the use of more sensitive staining methods and additional cultures. 

The reported incidence of TB in the general population in Colombia in 2010 was 25 cases/100000 inhabitants. Incidence rate ratios in prisons compared to the general population range from 8.5 to 20 (for Pereira and Medellin, respectively) [1]. 

There are several plausible explanations for the high incidence of TB in prisons: 1) there is no active case finding of latent TB infection (LTBI) and TB disease at the time of entry to prison and during incarceration. 2) Incarcerated individuals come from high risk-populations. 3) The growing prison population resulting in overcrowding and lessened personal space has been associated with the risk of TB infection[11]. 4) Logistical and financial (budget) constraints including frequent movement of prisoners between the prison system and the community, poor access to health care, and limited ability to attend local clinics or hospitals for security reasons[12], prevent proper treatment among inmates. 5) The country lacks a well-organized TB control program and offers ineffective health services, including lack of skilled and motivated manpower or adequate referral services, poorly controlled treatment services and supervision, and inadequate isolation rooms[12,13]. 6) Diagnostic algorithms are inaccurate, and adequate laboratory facilities are in short supply [12].

Among the diagnostic algorithms, one point that should be reevaluated is the duration of respiratory symptoms. The fact that this study found that 25% of TB cases had less than 15 days of respiratory symptoms (despite the absence of immunosuppression), of whom 66.7% were sputum smear-positive and presumably highly infectious, questions the validity of using the two-week criterion for screening individuals for TB in such a high-risk settings. There is a plethora of literature calling attention to the low sensitivity and specificity of the WHO criteria for diagnosing TB in HIV patients[3,14,15]. In the general population, the National Survey of TB prevalence in Vietnam showed that any type of cough (productive or dry) persisting more than 2 weeks was present in 54.9% of TB cases[16], and in a TB prevalence survey conducted in 2002 in two urban communities in Cape Town, South Africa, the sensitivity of cough ≥2 weeks was 54% (95% CI 31-76)[17]. Early diagnosis may truncate TB transmission, which is particularly important in prisons because of conditions such as overcrowded sleeping quarters, poor ventilation in prison cells, and congregated settings within the prisons[13], factors that operate together to facilitate the spread of TB. 

In addition to the duration of respiratory symptoms, other variables were identified as risk factors in this study and should be included when screening for TB: contact with a TB case, prior history of TB, and BMI <18 kg/m^2^. Similarly, in a study of a prison in Cameroon reported that 38% of inmates with pulmonary TB remained undetected, and incarceration in severely overcrowded cells, BMI <18.5 kg/m^2^, and previous TB treatment were associated with undetected TB cases[18]. In another report from a jail in Bangladesh[19], the main risk factors for TB were: exposure to TB patients, previous imprisonment, longer stay in prison, and BMI <18.5 kg/m^2^. In our study, all prisons were overcrowded, which made it impossible to include this variable in the multivariable analysis. Overcrowding may contribute to the high incidence we found as a risk factor throughout all prisons. We did not find an association between the time of imprisonment or history of prior incarceration. Some possible explanations for these findings include the young age of the inmates, recent incarceration (approximately 40% of people in these prisons had no sentence), and the frequent rotation of prisoners among national prisons for security reasons.

Although international and national guidelines recommend the use of culture plus acid-fast bacilli staining for the diagnosis of TB in prisons, these methods are not implemented in the prison system in Colombia. In this study, 23.6% of patients were sputum smear-negative with a positive sputum culture. Other studies have shown that culturing sputum samples increases the rate of detection of TB patients by 21 to 43.3% [3,14,19,20]. 

Moreover, culturing is important not only for diagnosis but also during follow-up. In this study, we found that the median time to culture conversion after the initiation of anti-TB treatment was 59 days, and the median time to sputum smear conversion was 33 days. These findings are in agreement with the results of other studies[5,6,21–23] conducted in the general population. Risk factors associated with a delayed sputum smear and culture conversion, including the AFB load at diagnosis, past history of TB, presence of cavitary disease[21,23] or bilateral radiological involvement[22], and mixed infection or re-infection during standard treatment[24].

We suggest that culture conversion should be used to determine the timing of the discontinuation of isolation during TB treatment. In addition, two findings support the use of culture methods during follow-up: 1) Several studies clearly document the transmission of TB from smear-negative patients[6,7,25,26], and 2) as discussed earlier, prisons have many factors acting together to allow for effective TB transmission[27]. These factors include prolonged exposure time (in the studied prisons, inmates are confined to cells for 12 to 14 hours per day), small room volume, high occupancy density (in this study, the mean area per person was less than 1.5 m^2^), low ventilation rate, and proximity to an index case.

The main limitation of this study was that not all patients had chest x-rays because this procedure was not available in any of the jails, obtaining a chest x-rays would have required the transfer to a hospital, which was not possible due to security concerns. Availability of chest x-rays may improve the ability to detect cases of active TB, suggesting that the incidence that we found could represent an underestimate. In addition, the lack of chest x-rays did not allow us to determine whether inmates with cavitary disease had longer times to sputum smear and culture conversion. Another limitation was that during the execution of the study, genotyping data was not available, hence we could not evaluate the relationship between TB cases.

This research reiterates the need to study TB in prisons and to implement an effective TB control program inside jails. This program should involve: 1) a political resolution; 2) permanent financing; 3) implementation and strengthening of the active case finding and DOTS-based program. We believe the aggressive case-finding intervention changed the incidence of TB in the prisons studied. This is reflected by the fact that the year prior to the study, 7 cases of TB had been diagnosed in one prison, and no cases had been reported in the other prisons. In the prisons studied, as well as in Colombia as a whole, no active case-finding is done; only passively. When we lead an active search, 48 cases of TB were identified in the four prisons in the first year. 4) A reliable information and data management system that allows for follow-up of the TB program; 5) engagement with the community (prisoners and their families); and 6) clinical experts to review complicated cases and protocols implemented in prisons. As O’Grady mentioned in 2011[28]: “A systematic approach to managing TB in prisons will lead to better optimized and cost-effective screening methods for TB, HIV and other infections, and ensure timely, rapid and accurate diagnosis and treatment of TB and HIV. Unless this is performed urgently, TB in prisons will remain a neglected global problem.” 
